# Supplementary material for: The Fourier-transform infrared spectroscopy-based method as a new typing tool for Candida parapsilosis clinical isolates
Source: Microbiol Spectr. 2023 Sep 11;11(5):e02388-23. doi: 10.1128/spectrum.02388-23 (PMC10580913; doi:10.1128/spectrum.02388-23)
Supplement: Table S1 — Characteristics of Candida parapsilosis (sensu stricto) isolates included in the study [file spectrum.02388-23-s0001.pdf]

TABLE S1 Characteristics of *Candida parapsilosis* (*sensu stricto*) isolates included in the study[a]

Isolate number	Isolate designation	Erg11p substitution(s)	Fluconazole susceptibility category[b]	Year of isolation	Hospital ward
1	CP06	Y132F	R	2014	Medicine
2	CP07	Y132F	R	2015	Medicine
3	CP08	Y132F	R	2015	Intensive care
4	CP09	Y132F	R	2015	Medicine
5	CP44	Y132F	R	2015	Medicine
6	CP45	Y132F	R	2015	Medicine
7	CP46	Y132F	R	2015	Medicine
8	CP01	Y132F	R	2016	Oncology
9	CP02	Y132F	R	2016	Medicine
10	CP03	Y132F	R	2016	Intensive care
11	CP26	Y132F	R	2016	Surgery
12	CP11	Y132F	R	2017	Intensive care
13	CP12	Y132F	R	2017	Medicine
14	CP13	Y132F	R	2017	Medicine
15	CP14	Y132F	R	2017	Medicine
16	CP15	Y132F	R	2017	Medicine
17	CP16	Y132F	R	2017	Surgery
18	CP18	Y132F	R	2017	Intensive care
19	CP19	Y132F	R	2017	Intensive care
20	CP21	Y132F	R	2017	Surgery
21	CP22	Y132F	R	2017	Medicine
22	CP23	Y132F	R	2017	Surgery
23	CP24	Y132F	R	2017	Medicine
24	CP25	Y132F	R	2017	Medicine
25	CP43	Y132F	R	2017	Surgery
26	CP05	Y132F	R	2018	Intensive care
27	CP27	Y132F	R	2018	Surgery
28	CP28	Y132F	R	2018	Oncology
29	CP29	Y132F	R	2018	Intensive care
30	CP30	Y132F	R	2018	Medicine
31	CP31	Y132F	R	2018	Medicine
32	CP37	Y132F	R	2018	Surgery
33	CP35	Y132F	R	2018	Intensive care
34	CP39	Y132F	R	2018	Medicine
35	CP41	Y132F	R	2018	Medicine
36	CP04	Y132F	R	2019	Medicine
37	CP47	Y132F	R	2019	Medicine
38	CP50	Y132F	R	2019	Intensive care
39	CP51	Y132F	R	2019	Medicine
**40**	**CP214 AR**	**Y132F**	**R**	**2022**	**Medicine**
41	CP42	Y132F/R391I	R	2016	Hematology
42	CP17	Y132F/R391I	R	2017	Medicine
43	CP20	Y132F/R391I	R	2017	Medicine
44	CP33	Y132F/R391I	R	2018	Medicine
45	CP34	Y132F/R391I	R	2018	Hematology
46	CP38	Y132F/R391I	R	2018	Medicine
47	CP40	Y132F/R391I	R	2018	Intensive care
48	CP49	Y132F/R391I	R	2019	Medicine
49	CP52	Y132F/R391I	R	2019	Medicine
**50**	**CP212 AR**	**Y132F/R391I**	**R**	**2022**	**Medicine**
51	CP630	None	S	2022	Medicine
52	CP631	None	S	2022	Medicine
53	CP632	None	S	2022	Medicine
54	CP641	None	S	2022	Medicine
55	CP645	None	S	2022	Medicine
56	CP655	None	S	2022	Medicine
57	CP657	None	S	2022	Medicine
58	CP662	None	S	2022	Medicine
**59**	**CP14 AR**	**None**	**S**	**2022**	**Medicine**

[a]Bold indicates isolates that had been recovered from hospital-acquired bloodstream infections in Arezzo, Italy. Except for those, all the isolates listed had been recovered from hospital-acquired bloodstream infections in Rome, Italy.

[b]Isolates were categorized as resistant (R) or susceptible (S) to fluconazole as detailed in the text.
